# Acute Parotitis after Lower Limb Amputation: A Case Report of a Rare Complication

**DOI:** 10.1155/2018/3714214

**Published:** 2018-03-15

**Authors:** Konstantinos Ioannis Avgerinos, Nikolaos Degermetzoglou, Sofia Theofanidou, Georgia Kritikou, Ioannis Bountouris

**Affiliations:** ^1^Vascular Surgery Clinic, 251 Hellenic Air Force General Hospital, Athens, Greece; ^2^Department of Medicine, Faculty of Health Sciences, Aristotle University of Thessaloniki, Thessaloniki, Greece; ^3^1st General Surgery, Vascular Surgery Department, Aristotle University of Thessaloniki, Thessaloniki, Greece; ^4^Department of Medicine, Faculty of Health Sciences, “Papageorgiou” General Hospital, Aristotle University of Thessaloniki, Thessaloniki, Greece; ^5^“Arogi” Rehabilitation Centre, Thessaloniki, Greece; ^6^Department of Economics Piraeus, University of Piraeus, Piraeus, Greece

## Abstract

**Background:**

Postoperative parotitis is a rare complication that occurs usually after abdominal surgery. Parotitis has never been described as a complication of vascular operations, in literature. In the present article, we describe a case of a postamputation parotitis along with its management and its possible pathogenesis.

**Case Report:**

An 83-year-old diabetic man was emergently admitted to hospital because of gangrene below the right ankle and sepsis. The patient underwent a lower limb amputation above the knee. On the 5th postoperative day, he was diagnosed with right parotitis probably because of dehydration, general anesthesia, and immunocompromisation. A CT scan confirmed the diagnosis. He received treatment with antibiotics and fluids. His condition gradually improved, and he was finally discharged on 15th postoperative day.

**Conclusions:**

Postoperative parotitis can possibly occur after any type of surgery including vascular. Clinicians should be aware of this complication although it is rare. Several risk factors such as dehydration, general anesthesia, drugs, immunocompromisation, head tilt during surgery, and stones in Stensen's duct may predispose to postoperative parotitis. Treatment consists of antibiotics and hydration.

## 1. Introduction

The viral or bacterial infection of the parotid gland is called parotitis and is one of the several types of sialadenitis [1]. Postoperative parotitis has been described as a complication of abdominal surgery [2]. The reported incidence in a large retrospective study was as low as 0.0028% [3]. Except from abdominal, acute parotitis has also been described in other types of operations such as those of neurosurgery field [4]. However, it has never been described as a postamputation complication in literature. In the present article, we report a postamputation case of parotitis and its management. We also investigate all possible risk factors for such a complication and we discuss the pathophysiology.

## 2. Case Report

An 83-year-old man with a history of peripheral artery disease and diabetes mellitus (DM) was admitted to hospital with gangrene below the level of ankle of the right foot. The patient was septic, with fever 37.6°C, WBC: 29.6 × 10^9^/L, Hct: 39.2%, CRP: 161 mg/L, and Cr: 5.2 *μ*mol/L. Diabetes mellitus had already resulted in complications such as diabetic retinopathy and nephropathy. Due to the severity of his condition, the patient emergently underwent amputation above the knee under general anesthesia. During the operation, the patient was transfused with one unit of concentrated red blood cells. The procedure was uneventful, and the patient woke up normally.

In the first four postoperative days, patient's wound was in good condition, and his renal function and inflammation markers were also improving. He also had no fever. However, he was considered to be severely malnourished because his albumin levels were 1.2 g/dL and had anasarca. The patient was started on a high-protein diet and intravenous antibiotics and was transfused with one unit of concentrated red blood cells during this time.

**Figure 1:**
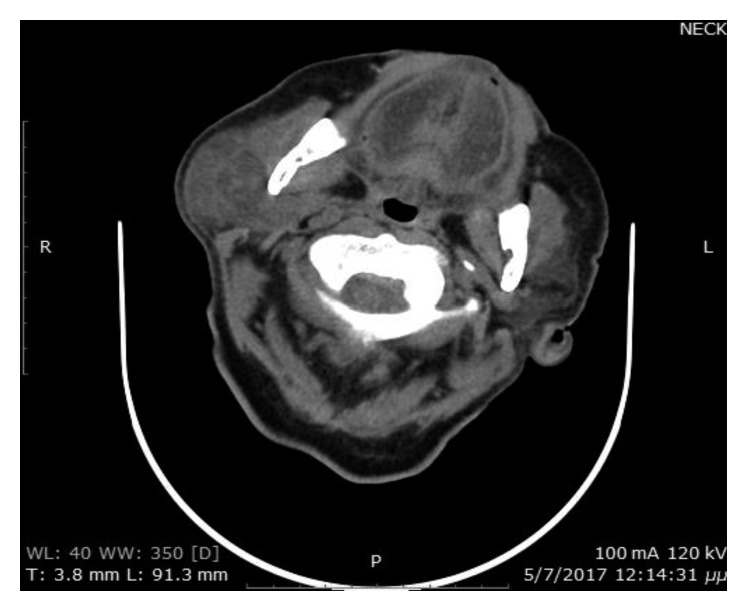


On the fifth postoperative day, the patient had right parotid gland swelling and fever up to 37.8°C, findings consistent with the diagnosis of parotitis. The patient was examined by an otolaryngologist, and a CT scan of the neck was scheduled. The imaging study confirmed the parotid gland swelling with hyperdense presentation, thickening of the fascia, and edema of the fat tissue. Additionally, there was enlargement of the unilateral masseter muscle. The parapharyngeal space was clear. Finally, no abscess formation was found (Figure 1).

Cultures from wound, inflamed Stensen's duct, and blood returned *Staphylococcus aureus* and *Pseudomonas aeruginosa*. The patient was started on metronidazole, piperacillin/tazobactam, and linezolid instead of clindamycin and ciprofloxacin that were previously given. Extra intravenous fluids were also given at 1000 mL/24 hr rate. During the next seven days, the patient's condition improved and the parotitis subsided. His laboratory findings were WBC 12.8 × 10^9^/L, CRP 31 mg/dL, Cr 2.7 *μ*mol/L, and Alb 1.8 g/dL. Two days later (postoperative day 15), the patient was discharged.

## 3. Ethical Considerations

Oral informed consent was obtained from the patient in order to publish data regarding his case. Also, the report of this man's case was approved by the institutional review board.

## 4. Discussion

Several factors have been identified as risk factors for postoperative acute parotitis [2]. Dehydration is a very important predisposing risk factor [5]. Our patient had already been in dehydration state even before surgery (on the admission day, he had urea 173 mg/dL), but he had to undergo an urgent amputation due to his septic state. Thus, there was no time for adequate fluid replacement. Manipulations of temporomandibular joint during general anesthesia may also be a risk factor [6]. Our patient's procedure was under general anesthesia too. Furthermore, the patient was malnourished, was diabetic for years, and had chronic renal failure. All these three factors are major contributors for postoperative acute parotitis, possibly because of the generalized immunosuppression state created by them [3].

In general, there are multiple risk factors that may play a role but were not identified in our case. Obstruction of Stensen's duct usually by a stone is usual [7]. Bad position during a surgery such as, for example, neck flexion or head tilt may also predispose to parotitis [4, 8]. In addition, several drugs such as morphine may be implicated in postoperative parotitis pathogenesis too [9, 10]. Morphine is thought to reduce the amylase secretion from parotid and the duct's smooth muscles activity [9, 10]. General conditions such as Sjogren's syndrome and hypothyroidism are rare risk factors [11].

Postoperative parotitis is a condition that usually appears during the 5th to 7th postoperative day, which was true for our case too [12]. The commonest offending pathogen is *Staphylococcus aureus* [11]. In our case, both *Staphylococcus aureus* and *Pseudomonas aeruginosa* were identified in the inflamed gland. Identical pathogens were found in blood and wound area, facts that support hematogenous spread from wound to parotid gland. Other commonly offending bacteria are streptococci species and *Haemophilus* influenza [11]. Treatment consists mainly of aggressive hydration and intravenous antibiotics [13].

## 5. Conclusion

The nosological entity of acute postoperative parotitis is usually described after abdominal surgery [5]. However, there are reports including other specialities' procedures such as those of neurosurgical nature [4]. In the present article, we reported the first case of acute parotitis after lower limb amputation. It is suspected that parotitis may occur after any possible surgery in a patient with several risk factors, both systematic and regional. Appropriate preoperative correction of these factors, if possible, may help reduce the risk of this complication. Clinicians should be aware of such a rare complication and whenever it occurs, antibiotics and hydration are the treatment of choice.

## References

[1] McQuone S. J. (1999). Acute viral and bacterial infections of the salivary glands. *Otolaryngologic Clinics of North America*.

[2] Sermoneta D., Lodoli C., Di Mugno M., De Cosmo G., Gui D. (2009). An unusual case of acute unilateral parotitis following abdominal surgery. Report of a case and review of the literature. *Annali Italiani di Chirurgia*.

[3] Belczak S. Q., Cleva R. D., Utiyama E. M., Cecconello I., Rasslan S., Parreira J. G. (2008). Acute postsurgical suppurative parotitis: current prevalence at Hospital das Clinicas, Sao Paulo University Medical School. *Revista do Instituto de Medicina Tropical de Sao Paulo*.

[4] Berker M., Sahin A., Aypar U., Ozgen T. (2004). Acute parotitis following sitting position neurosurgical procedures: review of five cases. *Journal of Neurosurgical Anesthesiology*.

[5] Raad I. I., Sabbagh M. F., Caranasos G. J. (1990). Acute bacterial sialadenitis: a study of 29 cases and review. *Reviews of Infectious Diseases*.

[6] Katayama T., Katou F., Motegi K. (1990). Unilateral parotid swelling after general anaesthesia. A case report. *Journal of Cranio-Maxillofacial Surgery*.

[7] Zou Z. J., Wang S. L., Zhu J. R., Wu Q. G., Yu S. F. (1992). Chronic obstructive parotitis. Report of ninety-two cases. *Oral Surgery, Oral Medicine, and Oral Pathology*.

[8] Postaci A., Aytac I., Oztekin C. V., Dikmen B. (2012). Acute unilateral parotid gland swelling after lateral decubitus position under general anesthesia. *Saudi Journal of Anaesthesia*.

[9] Miwa Y., Saeki M., Yamaji A., Maeda S., Saito K. (1996). Effect of morphine on secretion of amylase from isolated parotid acini. *Life Sciences*.

[10] Kenningham J. (2000). An unusual case of postoperative facial swelling. *Anaesthesia*.

[11] Wilson K. F., Meier J. D., Ward P. D. (2014). Salivary gland disorders. *American Family Physician*.

[12] Yonkers A. J., Krous H. F., Yarington C. T. (1972). Surgical parotitis. *Laryngoscope*.

[13] Brook I. (2003). Acute bacterial suppurative parotitis: microbiology and management. *Journal of Craniofacial Surgery*.

